# Evaluation of genetic susceptibility of common variants in *CACNA1D* with schizophrenia in Han Chinese

**DOI:** 10.1038/srep12935

**Published:** 2015-08-10

**Authors:** Fanglin Guan, Lu Li, Chuchu Qiao, Gang Chen, Tinglin Yan, Tao Li, Tianxiao Zhang, Xinshe Liu

**Affiliations:** 1 Key Laboratory of Environment and Genes Related to Diseases, Ministry of Education, Xi’an, China.; 2 Key Laboratory of National Ministry of Health for Forensic Sciences, School of Medicine & Forensics, Xi’an Jiaotong University, Xi’an, China.; 3 Department of Forensic Psychiatry, School of Medicine & Forensics, Xi’an Jiaotong University, Xi’an, China.; 4 Department of Psychiatry, Washington University in Saint Louis, MO, USA.; 5 Institute of Human Genomics & Forensic Sciences, Xi’an, China.

## Abstract

The heritability of schizophrenia (SCZ) has been estimated to be as high as 80%, suggesting that genetic factors may play an important role in the etiology of SCZ. Cav1.2 encoded by *CACNA1C* and Cav1.3 encoded by *CACNA1D* are dominant calcium channel-forming subunits of L-type Voltage-dependent Ca^2+^ channels, expressed in many types of neurons. The *CACNA1C* has been consistently found to be a risk gene for SCZ, but it is unknown for *CACNA1D*. To investigate the association of *CACNA1D* with SCZ, we designed a two-stage case-control study, including a testing set with 1117 cases and 1815 controls and a validation set with 1430 cases and 4295 controls in Han Chinese. A total of selected 97 tag single nucleotide polymorphisms (SNPs) in *CACNA1D* were genotyped, and single-SNP association, imputation analysis and gender-specific association analyses were performed in the two independent datasets. None was found to associate with SCZ. Further genotype and haplotype association analyses indicated a similar pattern in the two-stage study. Our findings suggested *CACNA1D* might not be a risk gene for SCZ in Han Chinese population, which add to the current state of knowledge regarding the susceptibility of *CACNA1D* to SCZ.

Schizophrenia (SCZ) is a devastating mental disorder which affects approximately 1% of the general population worldwide. Evidence from family, adoption, and twins studies supported high heritability in the development of SCZ (80%), which results from multiple loci with small effects^1^. New treatments are desperately needed to enhance the currently unacceptable rates of morbidity, non-response, and relapse. An important limiting factor is our lack of understanding of the molecular mechanism of system-level pathophysiology. Since the heritability of SCZ is very high, characterizing mechanisms of genetic risk is a promising route.

L-type voltage-gated Ca^2+^ channels (VGCCs), which comprises isoforms Cav1.1, Cav1.2, Cav1.3 and Cav1.4, contribute to some patterns of synaptic plasticity, including long-term potentiation and depression^2^,^3^. Whereas the expressions of Cav1.1 and Cav1.4 are restricted to skeletal muscle and retinal neurons respectively, Cav1.2 and Cav1.3 have a widely overlapping expression profile in the mammalian neurones^4^. They have previously been found in some brain areas involved in mood and anxiety (e.g. hippocampus, amygdala, prefrontal cortex cortex)^5^. Furthermore, they are both able to couple electrical activity to transcriptional regulation^6^,^7^. Cav1.3 channels activate at more negative voltages enabling them to modulate neuronal firing behavior and to serve pacemaker function in neurons^8^,^9^, which plays an important role in neuronal signaling and suggests that pharmacological modulation would also affect brain function to some extent. Therefore, these channels may be considered as potential new therapeutic targets for the treatment of some central nervous system (CNS) disorders.

Recent applications of genome-wide association studies (GWAS) and next generation sequencing have shown an overlap between the genetic variant that is susceptible to different psychiatric disorders^10^,^11^. Smoller *et al*. (2013) performed a meta-analysis of the GWAS data from 33,332 cases and 27,888 controls of European ancestory, which were distributed among the 5 major psychiatric disorders in Psychiatric Genomics Consortium (major depressive disorder, bipolar disorder, schizophrenia, autism spectrum disorders, and attention-deficit/hyperactivity disorder). In that study, they reported SNPs in 2 L-type voltage-gated calcium-channel subunits, CACNA1C and CACNB2, showed genome-wide significance in Europeans, which provided evidence that genetic variation in calcium channel signaling could increase the risk of these 5 neuropsychiatric disorders^12^. Accumulating evidence have been added for the association of CACNA1C with SCZ^13^,^14^,^15^,^16^,^17^,^18^, and CACNA1C is considered to be one of the most robust findings for genetic susceptibility of mental diseases^13^,^19^,^20^,^21^, implicating that variants within CACNA1C belong to a wide class of susceptibility factors shared across the psychiatric spectrum^12^,^22^,^23^.Based on the important modulatory role of L-type VGCCs (Cav1.2 and Cav1.3) in neurons, it would be very interesting to know relationship between SCZ and *CACNA1D* gene, encoding the alpha subunit of the L-type VGCC Cav1.3. To investigate the role of *CACNA1D* in SCZ susceptibility and its possible related biology function in SCZ, we designed a case-control study to identify whether selected common SNPs (97 tagSNPs) of *CACNA1D* were involved in SCZ by using two independent data sets of Han Chinese individuals.

## Results

### Allelic and genotypic association analysis

97 SNPs in the *CACNA1D* gene were genotyped in the testing dataset (1117 SCZ cases and 1815 controls). The allele and genotype distributions of all SNPs in both cases and controls, including the result of Hardy–Weinberg equilibrium (HWE) test, are shown in Table 1 and Table S1. They were highly polymorphic in both samples, and the allelic and the genotype distributions of them were all in HWE (P > 0.05).

We firstly conducted a single SNP association analysis in the testing dataset. When all the samples were considered, we observed potential associations for 6 SNPs (rs709323, rs3774458, rs1460118, rs3774530, rs3774601, rs3774605; p = 0.073731, p = 0.079330, p = 0.061349, p = 0.080180, p = 0.056828, p = 0.090101, respectively) (Table 1), although they were not statistically significant (0.1 > p ≥ 0.05). Genotype association analysis for 6 SNPs suggested a similar pattern with a trend of association in 3 SNPs (rs709323, rs3774458, rs3774601; p = 0.082293, p = 0.075241, p = 0.053895, respectively). The G allele of rs3774601 was more frequent in patients than that in controls (OR = 1.13, 95% CI: 1.00-1.28). The other 91SNPs did not differ significantly in both genotype and allele distributions. According to small effect sizes conferred by common alleles requiring the use of large samples, the overall state of a given SNP is best summarized by association-analysis of different populations. Therefore, we performed a single SNP association analysis for the above 6 SNPs in the validation dataset (1430 SCZ cases and 4295 controls). However, no potential or significant associations with SCZ were found in them (Table 1). To examine whether gender would play a role in the potential association as suggested in the testing dataset, we analyzed our data by separating males and females in the testing and validation datasets. Furthermore, we found no potential or significant associations with SCZ in females or males (Table S2).

### Imputation

**Figure 1:**
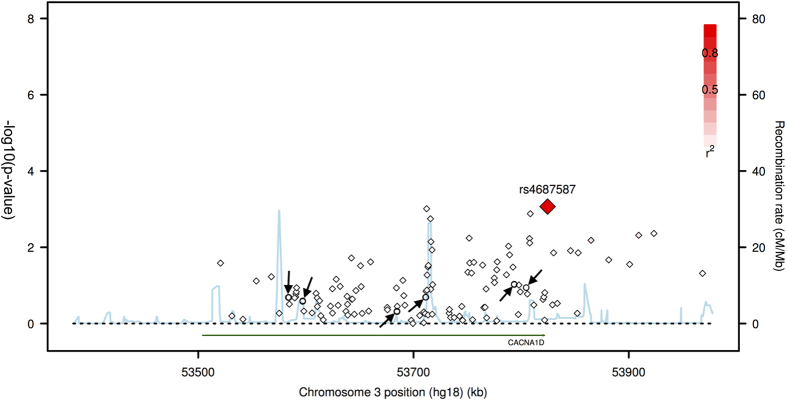
Regional association plot for both imputed and genotyped SNPs in HapMap CHB. This plot shows association results of imputed and genotyped SNPs within *CACNA1D* gene in Han Chinese cohort. The diamonds represent the imputed SNPs, and the circles represent the genotyped SNPs (marked by arrow), which are rs709323, rs3774458, rs1460118, rs3774530, rs3774601 and rs3774605 from left to right in order. The horizontal axis is the genomic context of the region studied (along with the genes). The left vertical axis represents negative logarithm of p-value and the right vertical axis is the recombination frequency of the region. Red diamond shows an imputed SNP, rs4687587, which has minimum p-value.

A total of 745 SNPs were successfully imputed. After applying the quality control procedure, 168 imputed SNPs were included in the association test. The full results of association test based on the typed and imputed SNPs were summarized in Table S3. The most significant SNP is an imputed SNP rs4687587 (p = 0.00085), however, considering the multiple comparison burden, this finding failed to pass Bonferroni correction threshold (0.05/130). The regional association plot based on HapMap CHB dataset was shown in Fig. 1.

### Haplotypic association analysis

**Figure 2:**
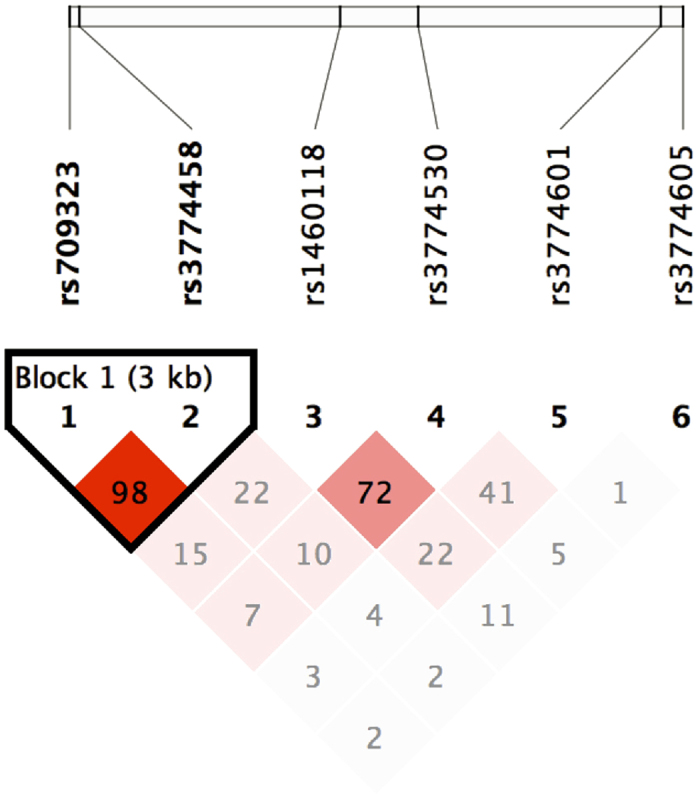
Estimation of LD between each pair of 6 SNPs in the validation dataset. LD structure (D’) between marker pairs was indicated by the shaded matrices.

To perform haplotype-based association analyses, we examined LD structure within the genotype data of 6 SNPs in validation dataset and identified one haplotype-block. Block 1 was 3 kb long and consisted of 2 SNPs (rs709323 and rs3774458) (Fig. 2). We next carried out haplotypic association analysis of the haplotype block, as was shown in Fig. 2. Tests of the two-marker haplotype association with the use of rs709323 and rs3774458 provided no evidence of association with SCZ (global p = 0.225 in GENECOUNTING and 0.324 in HAPLOSTATS) (Table 2). We also noticed that the haplotype frequencies estimated by two different programs, GENECOUNTING and Haplo Stats, were the same. Therefore, the potential bias reported by Curtis and Xu^24^ that minor differences in haplotype frequency estimates can produce very large differences in heterogeneity test statistics may not affect our analysis.

## Discussion

The predominant hypothesis in recent years has been that the genetic architecture of SCZ involves several common variants of small effects and possibly also rare variants with much larger effects^25^. It is reported that Cav1.2 and Cav1.3 have a broad and overlapping expression profile in the mammalian neuronal system^4^ and are both able to couple electrical activity to transcriptional regulation^6^,^7^. Moreover, as predominant isoforms of L-type VGCCs, they were found to be present in brain areas implicated in mood and anxiety (e.g. hippocampus, amygdala, prefrontal cortex)^5^,^26^. Previous studies have focused on the genetic variants within *CACNA1C* gene^19^,^20^,^27^. Recently, emerging evidence has supported that the role of variations within *CACNA1C* gene may also contribute to the risk of mental disorders^14^,^18^,^28^. In the present study, we evaluated the potential associations between genetic variations in the *CACNA1D* gene and the risk of SCZ.

We firstly dissected the association of *CACNA1D* polymorphisms with SCZ in two-stage case-control study of the Han Chinese population. We detected 97 tagSNPs of *CACNA1D* gene in the testing samples. Trends of associations were found in allele and genotype frequencies between patients and controls at 6 SNPs (rs709323, rs3774458, rs1460118, rs3774530, rs3774601 and rs3774605). Given that false association may arise from case–control study, because of the influence of small sample size, the second stage study in 5725 subjects was conducted as an effective approach to follow up the findings from the first stage study. However, no association was found in the samples, even though the statistical power of our study was enough to detect the different frequencies between patients and controls. Additionally, we identified an imputed SNP (rs4687587, p = 0.00085) with significant p value. However, considering the multiple comparison burden, the SNP failed to pass the Bonferroni correction. Imputation as a supplemental tool is widely used in common SNP based genetic association analysis, especially large scale analysis like GAWS. Although this method is proved to be powerful in some studies, the imputation accuracy is always a problem need to take care of and can be affected by several factors including the reference data utilized, the original data quality and density of the genotyped marker. To reduce the potential effects on association tests due to the inaccuracy of imputation, we applied several quality control criteria including the control of average maximum posterior probability. Our negative finding of imputation based analysis replicated the negative finding based on the genotyped markers. Furthermore, there was still no gender-specific association between these SNPs and SCZ. The ability to draw conclusions regarding associations based on the analysis of individual SNPs is limited^29^. Therefore, we performed haplotype analysis, which uses additional information on linkage between typed markers. The results of haplotype frequency estimation showed none of significant association with SCZ (p > 0.1, global permutation). To avoid the inaccuracy of haplotype frequency estimation which could lead to unreliable results, we used two different programs (GENECOUNTING and Haplo Stats) to conduct the haplotype association analyses, and the same results were obtained.

L-type VGCCs are found to involve in neuronal development and in the establishment of connectivity maintenance during development^30^. The increasing findings in genetic studies indicates that the CACNA1C gene is one of shared susceptibility factors for major psychiatric disorders and may have played an important role in the pathogenesis of these diseases at some level. However, the mechanisms illustrating how genetic variants within CACNA1C gene capture risk for developing psychiatric disorders are still unknown^14^,^18^,^28^. Ca^2+^ influx through L-type VGCCs is considered as a privileged signal, which transmits information of synaptic activation to the transcriptional machinery of the cell’s nucleus^31^. Some different signaling pathways, including activation of CREB (cAMP-response-element-binding protein), are involved in activity-dependent nuclear signaling via L-type VGCCs^31^. As an important signal integrator, CREB is responsible for critical central nervous system functions, such as learning, memory and depression-like effects, and both Cav1.2 and Cav1.3 can activate CREB in cultured neurons^32^. It has been reported that specific activation of Cav1.3 could induce depression-like behaviors^33^ and lead to activation of brain regions involved in anxiety and fear circuits^34^. Recently, *CACNA1D* genetic mutations have already been found to be involved in some neurological disorders including autism^35^,^36^,^37^,^38^ while a previous study indicated a possible role of Cav1.3 in the etiology of Parkinson’s disease^39^. When interpreting our results, we recognized that we designed the study based on the “Common Disease-Common Variant” hypothesis, and we had not sequenced the *CACNA1D* gene yet to completely evaluate the effect of rare variants on SCZ susceptibility. It could be possible that some rare variants might contribute to the risk of SCZ in a certain unpredicted way or in LD with other undiscovered markers involved with unknown machinery conferring the risk for SCZ. Thus, additional follow-up studies are required including high density mapping and targeted deep sequencing to undercover fundamental characteristics of pathogenic *CACNA1D* mutations and any potential association with SCZ.

A major limitation of the current study was that we did not perform further analyses for the possible risks of these SNPs that were involved in the clinic phenotype because of the lack of additional subgroups and clinic parameters of the high number of patients in the study. Moreover, although our moderate sample size was small compared with GWAS samples, it was larger than most of individual association studies. Total relatively, the statistical power to detect the moderate effect size for complex diseases such as schizophrenia was not strong, and all findings would need to be confirmed by further studies with enlarged sample size. Most recently, a large-scale GWAS17 has provided supportive evidence for our negative results and also implicated that rare variants in CACNA1D gene should be further studied and paid more attention by researchers. Therefore, as a two-stage designed case–control association study, the objective was reached and could be considered reasonable and reliable. Additionally, although we selected subjects with no migration history within the previous three generations to control population stratification caused by genetic factors, we did not focus much on other possible factors leading to population stratification; thus, hidden stratification interference could not be completely ruled out. All our interesting findings, therefore, should be considered preliminary, and these results should be interpreted with caution.

In summary, our studies did not support *CACNA1D* as a susceptible gene for SCZ in Han Chinese population. Our results contribute to a better understanding of the complex neurobiological mechanisms underlying SCZ and add to the current state of knowledge regarding the susceptibility of *CACNA1D* to SCZ. However, the present findings require replication to clarify the pathological mechanisms of L-type VGCCs for their functional roles in SCZ and to eventually make use in clinical practice. Future studies should not only include larger samples of patients and controls but also focus on the investigation and comparison of different patient samples that are more homogeneous in certain clinical phenotypes, which would be an important next step to demonstrate whether specific effects of *CACNA1D* genotype exist in subgroups of SCZ patients or not.

## Methods

### Participants

Two separate datasets were included in this study, and a two-stage approach was utilized for the discovery single marker analyses. Subjects containing 1117 SCZ cases (536 males, mean age = 36.0 ± 8.84; 581 females, mean age = 37.2 ± 8.54) and 1815 healthy controls (942 males, mean age = 35.7 ± 7.74; 873 females, mean age = 36.3 ± 7.46 were considered the testing set, while subjects containing 1430 SCZ cases (764 males, mean age = 34.4 ± 7.07; 666 females, mean age = 35.8 ± 6.81) as well as 4295 healthy controls (2280 males, mean age = 34.5 ± 7.02; 2015 females, mean age = 35.3 ± 6.95) were categorized as the validation set. All patients were recruited from the inpatient and outpatient clinical services of a psychiatric unit at Xi’an Mental Health Center, and all unrelated healthy controls from local volunteers.

All diagnoses were assigned by a standard procedure. After providing written informed consent, each subject was assessed with the Structured Clinical Interview for DSM-IV Axis I disorder (SCID), which was administered by two or more experienced psychiatrists. Standard diagnostic assessments were supplemented with clinical information obtained by a review of medical records and interviews with family informants. Detailed information on the onset and course of clinical disorders, the presence of personality disorders and mental retardation, and a brief description of the subject’s psychosocial and occupational functioning during the course of illness were presented to a consensus diagnostic group including at least three trained psychiatrists with DSM-IV (Diagnostic and statistical manual of mental disorders, 4th revision) diagnostic experience, as well as other trained SCID raters. All available information (personal history, hospital record, and family-history report) was used to arrive at a consensus DSM-IV diagnosis. Research subjects with substance-induced psychotic disorders, learning disabilities, head injuries, and other symptomatic psychoses were excluded from the present study. All subjects were recruited from the cities of Xi’an in Shaanxi Province. Based on self-report and medical records regarding their own and their paternal grandparents’ place of birth, we excluded anyone not born in Xi’an or whose families for three generations were not born in Xi’an. This study was performed in accordance with the ethical guidelines of the Declaration of Helsinki (version 2002) and was approved by the Xi’an Jiaotong University Ethics Committee. All of the participants have completed written informed consent forms.

### SNP selection and genotyping

We searched for all the SNPs with minor allele frequencies (MAF) ≥ 0.05 between 5 kb upstream and 5 kb downstream of *CACNA1D* gene in the HapMap CHB database by Haploview (Version 4.2), and found 153 SNPs in total. MAF ≥ 0.05 with pair-wise tagging and r^2^ ≥ 0.8^40^ were used as the criteria when selecting tagSNPs. Finally, we selected 97 tagSNPs covering the region of *CACNA1D* gene in the study.

Peripheral blood was drawn from a vein into a sterile tube containing ethylenediamine tetraacetic acid (EDTA). Plasma samples were stored at −80 °C. Genomic DNA was isolated from peripheral blood leukocytes according to the manufacturer’s protocol (Genomic DNA kit, Axygen Scientific Inc., California, USA). DNA was stored at −80 °C for SNP analysis. SNPs genotyping was performed using the Sequenom MassARRAY platform with the iPLEX GOLD chemistry (Sequenom, San Diego, CA, USA) following the manufacturer’s protocols. Polymerase chain reaction (PCR) primers and locus-specific extension primers were designed using MassARRAY Assay Design software package (v. 3.1). DNA template of 50 ng was used in each multiplexed PCR well. PCR products were treated with shrimp alkaline phosphatase (USB, Cleveland, OH, USA) before the iPLEX GOLD primer extension reaction. The single base extension products were desalted with SpectroCLEAN resin (Sequenom), and then an aliquot of 10 nL of the desalted product was spotted onto a 384-format SpectroCHIP with the MassARRAY Nanodispenser. Mass determination was done with the MALDI-TOF mass spectrometer. The MassARRAY Typer 4.0 software was employed for data acquisition. Because the final genotype call rate of each SNP was greater than 99.1% and the overall genotyping call rate was 99.6%, the reliability of further statistical analyses was ensured.

### Statistical analysis

Hardy–Weinberg equilibrium (HWE) was separately tested among the patient and control groups to examine the genotype distributions of each SNP mentioned above by using GENEPOP v4.0. Allelic and genotypic association tests were performed using CLUMP v2.4 with 10000 simulations, which employs an empirical Monte Carlo test of significance through simulation and does not require correction for multiple alleles^41^. To increase the density of the SNP markers in the validation dataset, we implemented imputation using genetic software IMPUT2^42^ with HapMap dataset from combined sample set as reference. The follow up association analysis was performed with software snptest^43^. We implemented frequentist association tests for each imputed SNPs and adding gender as a covariate. To ensure the accuracy of imputation, we only used the imputed SNPs with average maximum posterior probability larger than 0.5. For the power considerations, in the association test, we only included those SNPs with observed statistical information larger than 0.1 and with MAF larger than 0.01. The pair-wise linkage disequilibrium (LD) analysis was applied to detect the inter-marker relationship using D′and r^2^ values in the Haploview v4.2 software program. The haplotype frequencies were estimated using GENECOUNTING v2.2, and haplotypic association analysis was performed for the common haplotypes (frequency >0.01). In addition, a permutation algorithm was applied in the testing framework to find the maximum of the haplotype-specific score statistics and the associated p value for this maximum. The Haplo Stats package v1.4.4, which implements the methods of Schaid *et al*.^44^, was used for these analyses. To investigate the possible potential effect of gender for a trend of association of SNPs, the samples were stratified by gender. Power calculations are a fundamental component of the design of genetic association studies. We used Genetic Power Calculation (http://pngu.mgh.harvard.edu/~purcell/gpc/) to perform the power calculations. Our sample size had >80% power in the two-stage samples to detect a significant association at the false-positive rate of 5%, disease prevalence of 1%, MAF >5%, and a presumed odds ratio (OR) of 1.4 (results of general power analysis in Table S4 & S5).

## Additional Information

**How to cite this article**: Guan, F. *et al*. Evaluation of genetic susceptibility of common variants in *CACNA1D* with schizophrenia in Han Chinese. *Sci. Rep.* **5**, 12935; doi: 10.1038/srep12935 (2015).

## Supplementary Material

Supplementary Information

## Figures and Tables

**Table 1:** Allele and genotype frequency of single SNP association analysis in the testing and validation datasets.

Markers		The testing dataset									The validation dataset								
SNP ID/bp		Allele Freq.(%)		p-value^1^	Genotype Freq.(%)			p-value^1^	H-WE	OR^2^ 95%CI	Allele Freq.(%)		p-value^1^	Genotype Freq.(%)			p-value^1^	H-WE	OR^2^ 95%CI
rs709323		T	G		TT	GT	GG				T	G		TT	GT	GG			
SCZ	53,580, 457	1656(74.1)	578(25.9)	***0.073731***	620(55.5)	416(37.2)	81(7.25)	***0.082293***	0.331	1.12	2222(77.7)	638(22.3)	0.247525	868(60.7)	486(34.0)	76(5.31)	0.396040	0.460	1.07
CTR		2766(76.2)	864(23.8)		1047(57.7)	672(37.0)	96(5.29)		0.377	0.99–1.26	6769(78.8)	1821(21.2)		2667(62.1)	1435(33.4)	193(4.49)		0.999	0.94–1.18
rs3774458		G	A		GG	GA	AA				G	A		GG	GA	AA			
SCZ	53,583, 841	1784(79.9)	450(20.1)	***0.079330***	719(64.4)	346(31)	52(4.66)	***0.075241***	0.214	1.13	2369(82.8)	491(17.2)	0.277228	987(69.02)	395(27.6)	48(3.36)	0.316832	0.276	1.07
CTR		2966(81.7)	664(18.3)		1207(66.5)	552(30.4)	56(3.09)		0.458	0.99–1.29	7198(83.8)	1392(16.2)		3015(70.2)	1168(27.2)	112(2.61)		0.930	0.96–1.20
rs1460118		A	G		AA	AG	GG				A	G		AA	AG	GG			
SCZ	53,675, 824	1595(71.4)	639(28.6)	**0.061349**	573(51.3)	449(40.2)	95(8.5)	0.169869	0.597	1.12	2062(72.1)	798(27.9)	0.376238	748(52.31)	566(39.6)	116(8.11)	0.564356	0.539	1.05
CTR		2673(73.6)	957(26.4)		986(54.3)	701(38.6)	128(7.05)		0.823	0.99–1.26	6270(73.0)	2320(27.0)		2289(53.29)	1692(39.4)	314(7.31)		0.956	0.95–1.15
rs3774530		T	C		TT	TC	CC				T	C		TT	TC	CC			
SCZ	53,703, 343	1541(69)	693(31)	***0.080180***	526(47.1)	489(43.8)	102(9.13)	0.188375	0.443	1.09	1797(62.8)	1063(37.2)	0.178218	561(39.23)	675(47.2)	194(13.6)	0.207921	0.688	1.07
CTR		2582(71.1)	1048(28.9)		917(50.5)	748(41.2)	150(8.26)		0.884	0.98–1.24	5531(64.4)	3059(35.6)		1782(41.49)	1967(45.8)	546(12.7)		0.930	0.98–1.17
rs3774601		G	A		GG	GA	AA				G	A		GG	GA	AA			
SCZ	53,788, 866	1723(77.1)	511(22.9)	***0.056828***	656(58.7)	411(36.8)	50(4.48)	***0.053895***	0.152	1.13	2323(81.2)	537(18.8)	0.148515	937(65.52)	449(31.4)	44(3.08)	0.207921	0.266	1.10
CTR		2720(74.9)	910(25.1)		1024(56.4)	672(37)	119(6.56)		0.537	1.00–1.28	6855(79.8)	1735(20.2)		2736(63.7)	1383(32.2)	176(4.10)		0.941	0.98–1.22
rs3774605		C	T		CC	CT	TT				C	T		CC	CT	TT			
SCZ	53,796, 740	1932(86.5)	302(13.5)	***0.090101***	833(74.6)	266(23.8)	18(1.61)	0.156083	0.537	1.14	2591(90.6)	269(9.41)	0.178218	1177(82.31)	237(16.6)	16(1.12)	0.158416	0.299	1.11
CTR		3081(84.9)	549(15.1)		1312(72.3)	457(25.2)	46(2.53)		0.412	0.98–1.33	7705(89.7)	885(10.3)		3453(80.4)	799(18.6)	43(1.00)		0.669	0.96–1.28

SCZ: schizophrenia; CTR: control; CI: confidence interval; OR: odds ratio p values (p < 0.1) are in italic bold to indicate a trend of significant association.

^1^p-values of the normal chi-square statistics from Monte Carlo stimulation using CLUMP (T2).

^2^OR refers to risk allele odds ratio.

**Table 2:** Haplotypes frequency and association analysis in the validation dataset.

Haplotype	Haplotype	Genecounting (frequency %)				Haplo stats (frequency %)			
ID	rs709323-rs3774458	SCZ	CTR	p-value^1^	Global p^2^	SCZ	CTR	p-value^1^	Global p^2^
HAP1	TG	77.3	78.6	0.142	0.225	77.3	78.6	0.160	0.324
HAP2	GA	15.2	13.8	0.336		16.8	16.0	0.319	
HAP3	GG	5.55	5.20	0.477		5.53	5.26	0.499	

SCZ: schizophrenia; CTR: control.

^1^Based on 10000 permutations.

^2^Based on comparison of frequency distribution of all haplotypes for the combination of SNPs.
