# Supplementary material for: Evaluation of genetic susceptibility of common variants in CACNA1D with schizophrenia in Han Chinese
Source: Sci Rep. 2015 Aug 10;5:12935. doi: 10.1038/srep12935 (PMC4530443; doi:10.1038/srep12935)
Supplement: Supplementary Information [file srep12935-s1.pdf]

Title: Evaluation of genetic susceptibility of common variants in *CACNA1D* with schizophrenia in Han Chinese

Author names and affiliations: Fanglin Guan [a,e], Lu Li [b], Chuchu Qiao [b], Gang Chen [b], Tinglin Yan [b,c], Tao Li [b,c], Tianxiao Zhang [d] and Xinshe Liu [b,c]

[a] Key Laboratory of Environment and Genes Related to Diseases, Ministry of Education, Xi'an, China;

[b] Key Laboratory of National Ministry of Health for Forensic Sciences, School of Medicine & Forensics, Xi'an Jiaotong University, Xi'an, China

[c] Department of Forensic Psychiatry, School of Medicine & Forensics, Xi'an Jiaotong University, Xi'an, China;

[d] Department of Psychiatry, Washington University in Saint Louis, MO, USA;

[e] Institute of Human Genomics & Forensic Sciences, Xi'an, China.

Corresponding author: Xinshe Liu, Ph.D., M.D., Key Laboratory of National Ministry of Health for Forensic Sciences, School of Medicine, Xi'an Jiaotong University, Xi'an, China, 710061. Tel.: +86-29-82655475; Fax: +86-29-82655473, E-mail: lxins@mail.xjtu.edu.cn

TableS1 Allele and genotype frequency of single SNP association analysis in the testing dataset

Makers / SNP ID / bp		Allele Freq. (%)		p-value[1]	Genotype Freq. (%)			p-value[1]	H-W E p value
	rs312480	C	T		CC	CT	TT		
SCZ	53,495,113	1990(89.08)	244(10.92)	0.171346	889(79.59)	212(18.98)	16(1.43)	0.39283	0.411
CTR		3274(90.19)	356(9.81)		1479(81.49)	316(17.41)	20(1.1)		0.5
	rs219847	A	G		AA	AG	GG		
SCZ	53,499,800	1206(53.98)	1028(46.02)	0.361012	327(29.27)	552(49.42)	238(21.31)	0.629978	0.859
CTR		2004(55.21)	1626(44.79)		561(30.91)	882(48.6)	372(20.5)		0.457
	rs6763768	T	C		TT	TC	CC		
SCZ	53,503,852	1471(65.85)	763(34.15)	0.164871	327(29.27)	552(49.42)	238(21.31)	0.629978	0.859
CTR		2454(67.6)	1176(32.4)		561(30.91)	882(48.6)	372(20.5)		0.555
	rs3774419	G	A		GG	GA	AA		
SCZ	53,511,535	1699(76.05)	535(23.95)	0.464696	643(57.56)	413(36.97)	61(5.46)	0.75743	0.615
CTR		2730(75.21)	900(24.79)		1020(56.2)	690(38.02)	105(5.79)		0.408
	rs312487	C	T		CC	CT	TT		
SCZ	53,511,595	1145(51.25)	1089(48.75)	0.394802	290(25.96)	565(50.58)	262(23.46)	0.674224	0.682
CTR		1902(52.4)	1728(47.6)		497(27.38)	908(50.03)	410(22.59)		0.903
	rs17053118	C	T		CC	CT	TT		
SCZ	53,514,902	1829(81.87)	405(18.13)	0.193	748(66.97)	333(29.81)	36(3.22)	0.426531	0.886
CTR		3020(83.2)	610(16.8)		1256(69.2)	508(27.99)	51(2.81)		0.966
	rs312481	G	A		GG	GA	AA		
SCZ	53,517,875	1795(80.35)	439(19.65)	0.479508	719(64.37)	357(31.96)	41(3.67)	0.77304	0.686
CTR		2889(79.59)	741(20.41)		1145(63.09)	599(33)	71(3.91)		0.503
	rs979220	T	C		TT	TC	CC		
SCZ	53,522,196	2033(91)	201(9)	0.188377	926(82.9)	181(16.2)	10(0.9)	0.424737	0.726
CTR		3339(91.98)	291(8.02)		1537(84.68)	265(14.6)	13(0.72)		0.671
	rs3774424	G	T		GG	GT	TT		
SCZ	53,522,631	1890(84.6)	344(15.4)	0.690047	800(71.62)	290(25.96)	27(2.42)	0.923396	0.906
CTR		3085(84.99)	545(15.01)		1312(72.29)	461(25.4)	42(2.31)		0.841
	rs9311502	T	C		TT	TC	CC		
SCZ	53,526,294	1458(65.26)	776(34.74)	0.331342	471(42.17)	516(46.2)	130(11.64)	0.614988	0.529
CTR		2414(66.5)	1216(33.5)		797(43.91)	820(45.18)	198(10.91)		0.55
	rs17053141	G	A		GG	GA	AA		
SCZ	53,526,895	2088(93.46)	146(6.54)	0.150744	977(87.47)	134(12)	6(0.54)	0.227899	0.547
CTR		3426(94.38)	204(5.62)		1617(89.09)	192(10.58)	6(0.33)		0.906
	rs3774429	G	A		GG	GA	AA		
SCZ	53,532,379	1941(86.88)	293(13.12)	0.170041	840(75.2)	261(23.37)	16(1.43)	0.205917	0.399
CTR		3198(88.1)	432(11.9)		1412(77.8)	374(20.61)	29(1.6)		0.461
	rs3774437	C	T		CC	CT	TT		
SCZ	53,534,890	1942(86.93)	292(13.07)	0.316956	846(75.74)	250(22.38)	21(1.88)	0.572593	0.614

CTR		3122(86.01)	508(13.99)		1343(73.99)	436(24.02)	36(1.98)		0.929
	rs13078747	T	C		TT	TC	CC		
SCZ	53,539,490	1711(76.59)	523(23.41)	0.212894	650(58.19)	411(36.79)	56(5.01)	0.246743	0.384
CTR		2831(77.99)	799(22.01)		1109(61.1)	613(33.77)	93(5.12)		0.488
	rs3774439	T	A		TT	TA	AA		
SCZ	53,547,698	1758(78.69)	476(21.31)	0.315946	690(61.77)	378(33.84)	49(4.39)	0.525164	0.76
CTR		2816(77.58)	814(22.42)		1096(60.39)	624(34.38)	95(5.23)		0.614
	rs6764908	T	C		TT	TC	CC		
SCZ	53,549,441	1824(81.65)	410(18.35)	0.175897	748(66.97)	328(29.36)	41(3.67)	0.34544	0.5
CTR		3014(83.03)	616(16.97)		1251(68.93)	512(28.21)	52(2.87)		0.965
	rs2109182	C	T		CC	CT	TT		
SCZ	53,549,697	1902(85.14)	332(14.86)	0.171072	808(72.34)	286(25.6)	23(2.06)	0.364963	0.693
CTR		3137(86.42)	493(13.58)		1356(74.71)	425(23.42)	34(1.87)		0.917
	rs2359453	G	A		GG	GA	AA		
SCZ	53,558,837	1177(52.69)	1057(47.31)	0.254219	308(27.57)	561(50.22)	248(22.2)	0.517762	0.805
CTR		1968(54.21)	1662(45.79)		532(29.31)	904(49.81)	379(20.88)		0.889
	rs11715993	T	C		TT	TC	CC		
SCZ	53,565,945	1700(76.1)	534(23.9)	0.594052	649(58.1)	402(35.99)	66(5.91)	0.860411	0.72
CTR		2740(75.48)	890(24.52)		1036(57.08)	668(36.8)	111(6.12)		0.81
	rs17053186	C	T		CC	CT	TT		
SCZ	53,566,110	1918(85.85)	316(14.15)	0.50414	825(73.86)	268(23.99)	24(2.15)	0.798605	0.684
CTR		3139(86.47)	491(13.53)		1359(74.88)	421(23.2)	35(1.93)		0.719
	rs709318	C	T		CC	CT	TT		
SCZ	53,575,794	2034(91.05)	200(8.95)	0.343173	929(83.17)	176(15.76)	12(1.07)	0.595115	0.263
CTR		3278(90.3)	352(9.7)		1483(81.71)	312(17.19)	20(1.1)		0.432
	rs807193	A	G		AA	AG	GG		
SCZ	53,582,311	1430(64.01)	804(35.99)	0.340935	457(40.91)	516(46.2)	144(12.89)	0.63164	0.93
CTR		2368(65.23)	1262(34.77)		770(42.42)	828(45.62)	217(11.96)		0.806
	rs17053233	T	C		TT	TC	CC		
SCZ	53,590,523	1764(78.96)	470(21.04)	0.337886	699(62.58)	366(32.77)	52(4.66)	0.554554	0.645
CTR		2904(80)	726(20)		1160(63.91)	584(32.18)	71(3.91)		0.814
	rs6445588	C	T		CC	CT	TT		
SCZ	53,596,652	1587(71.04)	647(28.96)	0.251673	559(50.04)	469(41.99)	89(7.97)	0.510536	0.495
CTR		2629(72.42)	1001(27.58)		946(52.12)	737(40.61)	132(7.27)		0.479
	rs9866078	G	A		GG	GA	AA		
SCZ	53,601,415	1262(56.49)	972(43.51)	0.778689	354(31.69)	554(49.6)	209(18.71)	0.894498	0.765
CTR		2037(56.12)	1593(43.88)		574(31.63)	889(48.98)	352(19.39)		0.814
	rs3774473	G	A		GG	GA	AA		
SCZ	53,604,824	1804(80.75)	430(19.25)	0.681676	731(65.44)	342(30.62)	44(3.94)	0.662441	0.614
CTR		2947(81.18)	683(18.82)		1192(65.67)	563(31.02)	60(3.31)		0.513
	rs13063116	A	G		AA	AG	GG		
SCZ	53,615,650	1841(82.41)	393(17.59)	0.504351	762(68.22)	317(28.38)	38(3.4)	0.649218	0.479

CTR		3016(83.09)	614(16.91)		1252(68.98)	512(28.21)	51(2.81)		0.877
	rs898413	A	G		AA	AG	GG		
SCZ	53,617,389	1931(86.44)	303(13.56)	0.382238	838(75.02)	255(22.83)	24(2.15)	0.373273	0.378
CTR		3108(85.62)	522(14.38)		1327(73.11)	454(25.01)	34(1.87)		0.501
	rs3796345	C	T		CC	CT	TT		
SCZ	53,619,125	1561(69.87)	673(30.13)	0.284616	547(48.97)	467(41.81)	103(9.22)	0.537793	0.817
CTR		2584(71.18)	1046(28.82)		918(50.58)	748(41.21)	149(8.21)		0.845
	rs3774480	C	T		CC	CT	TT		
SCZ	53,620,866	1633(73.1)	601(26.9)	0.809772	591(52.91)	451(40.38)	75(6.71)	0.955272	0.374
CTR		2643(72.81)	987(27.19)		955(52.62)	733(40.39)	127(7)		0.394
	rs12493744	T	C		TT	TC	CC		
SCZ	53,626,003	1207(54.03)	1027(45.97)	0.305686	322(28.83)	563(50.4)	232(20.77)	0.549464	0.625
CTR		2011(55.4)	1619(44.6)		557(30.69)	897(49.42)	361(19.89)		0.997
	rs3774491	C	G		CC	CG	GG		
SCZ	53,629,808	1628(72.87)	606(27.13)	0.290209	596(53.36)	436(39.03)	85(7.61)	0.54837	0.671
CTR		2599(71.6)	1031(28.4)		931(51.29)	737(40.61)	147(8.1)		0.946
	rs6802110	G	A		GG	GA	AA		
SCZ	53,633,205	1972(88.27)	262(11.73)	0.133659	871(77.98)	230(20.59)	16(1.43)	0.326868	0.854
CTR		3250(89.53)	380(10.47)		1456(80.22)	338(18.62)	21(1.16)		0.781
	rs1380606	C	T		CC	CT	TT		
SCZ	53,634,105	1820(81.47)	414(18.53)	0.47597	737(65.98)	346(30.98)	34(3.04)	0.69388	0.387
CTR		2930(80.72)	700(19.28)		1180(65.01)	570(31.4)	65(3.58)		0.707
	rs6786135	T	C		TT	TC	CC		
SCZ	53,635,938	1609(72.02)	625(27.98)	0.303705	581(52.01)	447(40.02)	89(7.97)	0.579152	0.815
CTR		2569(70.77)	1061(29.23)		909(50.08)	751(41.38)	155(8.54)		0.995
	rs2077167	C	T		CC	CT	TT		
SCZ	53,637,392	1273(56.98)	961(43.02)	0.389335	355(31.78)	563(50.4)	199(17.82)	0.3362	0.347
CTR		2110(58.13)	1520(41.87)		621(34.21)	868(47.82)	326(17.96)		0.454
	rs6803827	G	A		GG	GA	AA		
SCZ	53,642,306	1503(67.28)	731(32.72)	0.59832	508(45.48)	487(43.6)	122(10.92)	0.868129	0.744
CTR		2418(66.61)	1212(33.39)		808(44.52)	802(44.19)	205(11.29)		0.778
	rs2276836	C	T		CC	CT	TT		
SCZ	53,651,012	1161(51.97)	1073(48.03)	0.350473	298(26.68)	565(50.58)	254(22.74)	0.609964	0.659
CTR		1932(53.22)	1698(46.78)		514(28.32)	904(49.81)	397(21.87)		0.99
	rs3774519	T	G		TT	TG	GG		
SCZ	53,661,215	1955(87.51)	279(12.49)	0.354773	857(76.72)	241(21.58)	19(1.7)	0.624568	0.666
CTR		3206(88.32)	424(11.68)		1416(78.02)	374(20.61)	25(1.38)		0.957
	rs3774523	T	C		TT	TC	CC		
SCZ	53,666,733	1798(80.48)	436(19.52)	0.707417	727(65.09)	344(30.8)	46(4.12)	0.710416	0.511
CTR		2936(80.88)	694(19.12)		1185(65.29)	566(31.18)	64(3.53)		0.722
	rs2633707	C	G		CC	CG	GG		
SCZ	53,675,055	1719(76.95)	515(23.05)	0.287939	665(59.53)	389(34.83)	63(5.64)	0.554793	0.539

CTR		2749(75.73)	881(24.27)		1044(57.52)	661(36.42)	110(6.06)		0.693
	rs12492502	A	G		AA	AG	GG		
SCZ	53,681,047	1250(55.95)	984(44.05)	0.91604	347(31.07)	556(49.78)	214(19.16)	0.864254	0.742
CTR		2026(55.81)	1604(44.19)		570(31.4)	886(48.82)	359(19.78)		0.66
	rs898411	T	C		TT	TC	CC		
SCZ	53,682,885	1580(70.73)	654(29.27)	0.269071	562(50.31)	456(40.82)	99(8.86)	0.546428	0.636
CTR		2616(72.07)	1014(27.93)		947(52.18)	722(39.78)	146(8.04)		0.61
	rs11130377	T	C		TT	TC	CC		
SCZ	53,688,847	1347(60.3)	887(39.7)	0.822481	403(36.08)	541(48.43)	173(15.49)	0.970151	0.699
CTR		2178(60)	1452(40)		650(35.81)	878(48.37)	287(15.81)		0.739
	rs1380608	A	G		AA	AG	GG		
SCZ	53,690,343	1925(86.17)	309(13.83)	0.212745	834(74.66)	257(23.01)	26(2.33)	0.279825	0.245
CTR		3085(84.99)	545(15.01)		1310(72.18)	465(25.62)	40(2.2)		0.867
	rs9841978	G	A		GG	GA	AA		
SCZ	53,696,708	1582(70.81)	652(29.19)	0.796408	554(49.6)	474(42.44)	89(7.97)	0.802103	0.374
CTR		2582(71.13)	1048(28.87)		917(50.52)	748(41.21)	150(8.26)		0.884
	rs7340705	T	C		TT	TC	CC		
SCZ	53,700,416	1215(54.39)	1019(45.61)	0.325205	324(29.01)	567(50.76)	226(20.23)	0.574107	0.44
CTR		2022(55.7)	1608(44.3)		559(30.8)	904(49.81)	352(19.39)		0.693
	rs6445597	G	A		GG	GA	AA		
SCZ	53,700,504	1386(62.04)	848(37.96)	0.847872	435(38.94)	516(46.2)	166(14.86)	0.912998	0.521
CTR		2243(61.79)	1387(38.21)		695(38.29)	853(47)	267(14.71)		0.841
	rs9830632	A	G		AA	AG	GG		
SCZ	53,701,739	1877(84.02)	357(15.98)	0.305352	792(70.9)	293(26.23)	32(2.86)	0.594443	0.439
CTR		3086(85.01)	544(14.99)		1316(72.51)	454(25.01)	45(2.48)		0.435
	rs3774529	G	T		GG	GT	TT		
SCZ	53,703,134	1898(84.96)	336(15.04)	0.380538	805(72.07)	288(25.78)	24(2.15)	0.640292	0.767
CTR		3053(84.1)	577(15.9)		1285(70.8)	483(26.61)	47(2.59)		0.841
	rs3774533	A	G		AA	AG	GG		
SCZ	53,704,293	1427(63.88)	807(36.12)	0.388287	451(40.38)	525(47)	141(12.62)	0.683596	0.537
CTR		2359(64.99)	1271(35.01)		760(41.87)	839(46.23)	216(11.9)		0.502
	rs2633731	C	T		CC	CT	TT		
SCZ	53,704,397	1798(80.48)	436(19.52)	0.277249	726(65)	346(30.98)	45(4.03)	0.51404	0.64
CTR		2963(81.63)	667(18.37)		1209(66.61)	545(30.03)	61(3.36)		0.965
	rs3821857	T	C		TT	TC	CC		
SCZ	53,704,433	1823(81.6)	411(18.4)	0.394574	746(66.79)	331(29.63)	40(3.58)	0.638917	0.662
CTR		2994(82.48)	636(17.52)		1234(67.99)	526(28.98)	55(3.03)		0.907
	rs2633730	C	T		CC	CT	TT		
SCZ	53,706,672	1183(52.95)	1051(47.05)	0.30662	308(27.57)	567(50.76)	242(21.67)	0.567338	0.53
CTR		1972(54.33)	1658(45.67)		532(29.31)	908(50.03)	375(20.66)		0.73
	rs2633728	C	G		CC	CG	GG		
SCZ	53,706,912	1854(82.99)	380(17.01)	0.325993	774(69.29)	306(27.39)	37(3.31)	0.596633	0.321

CTR		2976(81.98)	654(18.02)		1225(67.49)	526(28.98)	64(3.53)		0.419
	rs3774541	G	A		GG	GA	AA		
SCZ	53,707,105	1608(71.98)	626(28.02)	0.229332	574(51.39)	460(41.18)	83(7.43)	0.474874	0.485
CTR		2665(73.42)	965(26.58)		973(53.61)	719(39.61)	123(6.78)		0.526
	rs2680661	T	G		TT	TG	GG		
SCZ	53,708,121	1227(54.92)	1007(45.08)	0.430219	333(29.81)	561(50.22)	223(19.96)	0.714309	0.632
CTR		2032(55.98)	1598(44.02)		566(31.18)	900(49.59)	349(19.23)		0.794
	rs2612015	T	G		TT	TG	GG		
SCZ	53,708,304	2011(90.02)	223(9.98)	0.794922	904(80.93)	203(18.17)	10(0.9)	0.86389	0.707
CTR		3260(89.81)	370(10.19)		1465(80.72)	330(18.18)	20(1.1)		0.769
	rs6445598	A	T		AA	AT	TT		
SCZ	53,708,709	1763(78.92)	471(21.08)	0.190476	694(62.13)	375(33.57)	48(4.3)	0.414344	0.767
CTR		2916(80.33)	714(19.67)		1171(64.52)	574(31.63)	70(3.86)		0.974
	rs3821858	T	C		TT	TC	CC		
SCZ	53,713,653	1950(87.29)	284(12.71)	0.125022	850(76.1)	250(22.38)	17(1.52)	0.303802	0.777
CTR		3217(88.62)	413(11.38)		1425(78.51)	367(20.22)	23(1.27)		0.908
	rs2070617	G	A		GG	GA	AA		
SCZ	53,724,022	1329(59.49)	905(40.51)	0.498884	391(35)	547(48.97)	179(16.03)	0.781694	0.593
CTR		2127(58.6)	1503(41.4)		619(34.1)	889(48.98)	307(16.91)		0.687
	rs7638857	C	A		CC	CA	AA		
SCZ	53,724,296	1592(71.26)	642(28.74)	0.233121	565(50.58)	462(41.36)	90(8.06)	0.467973	0.743
CTR		2639(72.7)	991(27.3)		960(52.89)	719(39.61)	136(7.49)		0.931
	rs17053417	G	A		GG	GA	AA		
SCZ	53,725,362	1971(88.23)	263(11.77)	0.404589	868(77.71)	235(21.04)	14(1.25)	0.647895	0.67
CTR		3176(87.49)	454(12.51)		1390(76.58)	396(21.82)	29(1.6)		0.896
	rs1020819	C	A		CC	CA	AA		
SCZ	53,728,686	1595(71.4)	639(28.6)	0.283622	574(51.39)	447(40.02)	96(8.59)	0.440466	0.499
CTR		2544(70.08)	1086(29.92)		889(48.98)	766(42.2)	160(8.82)		0.784
	rs17030975	C	T		CC	CT	TT		
SCZ	53,734,178	1394(62.4)	840(37.6)	0.539915	441(39.48)	512(45.84)	164(14.68)	0.665986	0.438
CTR		2294(63.2)	1336(36.8)		724(39.89)	846(46.61)	245(13.5)		0.931
	rs1045958	C	T		CC	CT	TT		
SCZ	53,735,452	1614(72.25)	620(27.75)	0.135542	588(52.64)	438(39.21)	91(8.15)	0.258517	0.459
CTR		2687(74.02)	943(25.98)		993(54.71)	701(38.62)	121(6.67)		0.856
	rs11915963	T	C		TT	TC	CC		
SCZ	53,737,887	1997(89.39)	237(10.61)	0.455105	890(79.68)	217(19.43)	10(0.9)	0.34406	0.417
CTR		3267(90)	363(10)		1474(81.21)	319(17.58)	22(1.21)		0.315
	rs2612027	A	G		AA	AG	GG		
SCZ	53,742,651	1206(53.98)	1028(46.02)	0.14714	317(28.38)	572(51.21)	228(20.41)	0.199044	0.305
CTR		2030(55.92)	1600(44.08)		572(31.52)	886(48.82)	357(19.67)		0.676
	rs2680648	T	C		TT	TC	CC		
SCZ	53,743,149	1669(74.71)	565(25.29)	0.440916	619(55.42)	431(38.59)	67(6)	0.624231	0.481

CTR		2679(73.8)	951(26.2)		989(54.49)	701(38.62)	125(6.89)		0.959
	rs2253795	G	T		GG	GT	TT		
SCZ	53,744,848	1888(84.51)	346(15.49)	0.115448	801(71.71)	286(25.6)	30(2.69)	0.285943	0.464
CTR		3122(86.01)	508(13.99)		1345(74.1)	432(23.8)	38(2.09)		0.632
	rs17053459	A	G		AA	AG	GG		
SCZ	53,746,967	2037(91.18)	197(8.82)	0.173832	929(83.17)	179(16.03)	9(0.81)	0.396657	0.907
CTR		3271(90.11)	359(9.89)		1474(81.21)	323(17.8)	18(0.99)		0.948
	rs3774560	T	C		TT	TC	CC		
SCZ	53,751,376	2000(89.53)	234(10.47)	0.174597	897(80.3)	206(18.44)	14(1.25)	0.389449	0.578
CTR		3208(88.37)	422(11.63)		1419(78.18)	370(20.39)	26(1.43)		0.737
	rs17053472	A	C		AA	AC	CC		
SCZ	53,754,705	2036(91.14)	198(8.86)	0.287497	930(83.26)	176(15.76)	11(0.98)	0.506773	0.41
CTR		3278(90.3)	352(9.7)		1481(81.6)	316(17.41)	18(0.99)		0.802
	rs3774563	A	G		AA	AG	GG		
SCZ	53,756,249	1852(82.9)	382(17.1)	0.367205	773(69.2)	306(27.39)	38(3.4)	0.594272	0.26
CTR		3042(83.8)	588(16.2)		1278(70.41)	486(26.78)	51(2.81)		0.559
	rs3796347	G	A		GG	GA	AA		
SCZ	53,758,863	1807(80.89)	427(19.11)	0.152984	729(65.26)	349(31.24)	39(3.49)	0.340651	0.726
CTR		2990(82.37)	640(17.63)		1232(67.88)	526(28.98)	57(3.14)		0.925
	rs3774573	T	C		TT	TC	CC		
SCZ	53,765,933	1853(82.95)	381(17.05)	0.348147	771(69.02)	311(27.84)	35(3.13)	0.642204	0.595
CTR		2976(81.98)	654(18.02)		1223(67.38)	530(29.2)	62(3.42)		0.624
	rs1109959	T	C		TT	TC	CC		
SCZ	53,766,195	1884(84.33)	350(15.67)	0.120806	798(71.44)	288(25.78)	31(2.78)	0.277038	0.417
CTR		3115(85.81)	515(14.19)		1338(73.72)	439(24.19)	38(2.09)		0.777
	rs3821865	T	C		TT	TC	CC		
SCZ	53,768,268	1446(64.73)	788(35.27)	0.569776	475(42.52)	496(44.4)	146(13.07)	0.615609	0.357
CTR		2323(63.99)	1307(36.01)		742(40.88)	839(46.23)	234(12.89)		0.895
	rs3774581	A	G		AA	AG	GG		
SCZ	53,768,721	1331(59.58)	903(40.42)	0.184596	394(35.27)	543(48.61)	180(16.11)	0.408563	0.756
CTR		2226(61.32)	1404(38.68)		681(37.52)	864(47.6)	270(14.88)		0.881
	rs7617553	G	A		GG	GA	AA		
SCZ	53,779,205	1453(65.04)	781(34.96)	0.411795	474(42.44)	505(45.21)	138(12.35)	0.707901	0.845
CTR		2399(66.09)	1231(33.91)		793(43.69)	813(44.79)	209(11.52)		0.977
	rs11707420	G	A		GG	GA	AA		
SCZ	53,781,838	1653(73.99)	581(26.01)	0.248435	613(54.88)	427(38.23)	77(6.89)	0.505318	0.822
CTR		2636(72.62)	994(27.38)		957(52.73)	722(39.78)	136(7.49)		0.991
	rs3774589	G	T		GG	GT	TT		
SCZ	53,783,331	1485(66.47)	749(33.53)	0.503362	489(43.78)	507(45.39)	121(10.83)	0.757562	0.54
CTR		2382(65.62)	1248(34.38)		779(42.92)	824(45.4)	212(11.68)		0.792
	rs3774604	C	T		CC	CT	TT		
SCZ	53,790,109	1788(80.04)	446(19.96)	0.357732	712(63.74)	364(32.59)	41(3.67)	0.588168	0.51

CTR		2869(79.04)	761(20.96)		1133(62.42)	603(33.22)	79(4.35)		0.913
	rs3774609	T	G		TT	TG	GG		
SCZ	53,798,876	1386(62.04)	848(37.96)	0.299791	427(38.23)	532(47.63)	158(14.15)	0.509647	0.708
CTR		2301(63.39)	1329(36.61)		733(40.39)	835(46.01)	247(13.61)		0.707
	rs3796349	A	G		AA	AG	GG		
SCZ	53,799,482	1723(77.13)	511(22.87)	0.285156	667(59.71)	389(34.83)	61(5.46)	0.528885	0.664
CTR		2843(78.32)	787(21.68)		1113(61.32)	617(33.99)	85(4.68)		0.966
	rs2359133	C	G		CC	CG	GG		
SCZ	53,802,715	1654(74.04)	580(25.96)	0.347325	609(54.52)	436(39.03)	72(6.45)	0.575839	0.608
CTR		2647(72.92)	983(27.08)		966(53.22)	715(39.39)	134(7.38)		0.915
	rs3774611	G	A		GG	GA	AA		
SCZ	53,806,638	1183(52.95)	1051(47.05)	0.124304	308(27.57)	567(50.76)	242(21.67)	0.249082	0.53
CTR		1997(55.01)	1633(44.99)		552(30.41)	893(49.2)	370(20.39)		0.799
	rs10154841	C	T		CC	CT	TT		
SCZ	53,811,268	1826(81.74)	408(18.26)	0.152133	743(66.52)	340(30.44)	34(3.04)	0.317411	0.514
CTR		3020(83.2)	610(16.8)		1256(69.2)	508(27.99)	51(2.81)		0.966
	rs10490844	A	G		AA	AG	GG		
SCZ	53,812,050	1878(84.06)	356(15.94)	0.122258	787(70.46)	304(27.22)	26(2.33)	0.247641	0.597
CTR		2995(82.51)	635(17.49)		1238(68.21)	519(28.6)	58(3.2)		0.689
	rs877483	C	T		CC	CT	TT		
SCZ	53,812,714	1962(87.82)	272(12.18)	0.178846	865(77.44)	232(20.77)	20(1.79)	0.20131	0.336
CTR		3144(86.61)	486(13.39)		1358(74.82)	428(23.58)	29(1.6)		0.474
	rs877484	G	A		GG	GA	AA		
SCZ	53,812,897	1734(77.62)	500(22.38)	0.217942	675(60.43)	384(34.38)	58(5.19)	0.467788	0.724
CTR		2867(78.98)	763(21.02)		1134(62.48)	599(33)	82(4.52)		0.798

SCZ: schizophrenia; CTR: control

1. p-values of the normal chi-square statistics from Monte Carlo stimulation using CLUMP (T2).

TableS2 Gender specific allele and genotype association analysis in the testing and validation datasets

Markers		The testing dataset								The validation dataset							
SNP ID		Allele Freq. (%)		p-value[1]	Genotype Freq. (%)			p-value[1]	H-WE p-value	Allele Freq. (%)		p-value[1]	Genotype Freq. (%)			p-value[1]	H-WE p-value
rs709323		T	G		TT	GT	GG			T	G		TT	GT	GG		
F	SCZ	865(74.44)	297(25.56)	0.338366	325(55.94)	215(37.01)	41(7.06)	0.417513	0.507	1014(76.13)	318(23.87)	0.118812	386(57.96)	242(36.34)	38(5.71)	0.435644	0.993
	CTR	1327(76)	419(24)		501(57.39)	325(37.23)	47(5.38)		0.543	3139(77.89)	891(22.11)		1221(60.6)	697(34.59)	97(4.81)		0.847
M	SCZ	791(73.79)	281(26.21)	0.115449	295(55.04)	201(37.5)	40(7.46)	0.177906	0.479	1208(79.06)	320(20.94)	0.643564	482(63.09)	244(31.94)	38(4.97)	0.673267	0.326
	CTR	1439(76.38)	445(23.62)		546(57.96)	347(36.84)	49(5.2)		0.521	3630(79.61)	930(20.39)		1446(63.42)	738(32.37)	96(4.21)		0.881
rs3774458		G	A		GG	GA	AA			G	A			GG	GA	AA	
F	SCZ	921(79.26)	241(20.74)	0.193250	368(63.34)	185(31.84)	28(4.82)	0.260661	0.448	1099(82.51)	233(17.49)	0.128713	455(68.32)	189(28.38)	22(3.3)	0.386139	0.663
	CTR	1418(81.21)	328(18.79)		573(65.64)	272(31.16)	28(3.21)		0.533	3390(84.12)	640(15.88)		1425(70.72)	540(26.8)	50(2.48)		0.891
M	SCZ	863(80.5)	209(19.5)	0.262687	351(65.49)	161(30.04)	24(4.48)	0.304203	0.318	1270(83.12)	258(16.88)	0.683168	532(69.63)	206(26.96)	26(3.4)	0.623762	0.277
	CTR	1548(82.17)	336(17.83)		634(67.3)	280(29.72)	28(2.97)		0.663	3808(83.51)	752(16.49)		1590(69.74)	628(27.54)	62(2.72)		0.999
rs1460118		A	G		AA	AG	GG			A	G			AA	AG	GG	
F	SCZ	836(71.94)	326(28.06)	0.184264	306(52.67)	224(38.55)	51(8.78)	0.410301	0.279	964(72.37)	368(27.63)	0.316832	354(53.15)	256(38.44)	56(8.41)	0.396040	0.317
	CTR	1295(74.17)	451(25.83)		485(55.56)	325(37.23)	63(7.22)		0.401	2970(73.7)	1060(26.3)		1094(54.29)	782(38.81)	139(6.9)		0.963
M	SCZ	759(70.8)	313(29.2)	0.171790	267(49.81)	225(41.98)	44(8.21)	0.387195	0.723	1098(71.86)	430(28.14)	0.722772	394(51.57)	310(40.58)	60(7.85)	0.940594	0.928
	CTR	1378(73.14)	506(26.86)		501(53.18)	376(39.92)	65(6.9)		0.625	3300(72.37)	1260(27.63)		1195(52.41)	910(39.91)	175(7.68)		0.923
rs3774530		T	C		TT	TC	CC			T	C			TT	TC	CC	
F	SCZ	795(68.42)	367(31.58)	0.286259	269(46.3)	257(44.23)	55(9.47)	0.492239	0.570	821(61.64)	511(38.36)	0.168317	248(37.24)	325(48.8)	93(13.96)	0.326733	0.411
	CTR	1227(70.27)	519(29.73)		432(49.48)	363(41.58)	78(8.93)		0.889	2559(63.5)	1471(36.5)		812(40.3)	935(46.4)	268(13.3)		0.964
M	SCZ	746(69.59)	326(30.41)	0.178907	257(47.95)	232(43.28)	47(8.77)	0.393473	0.600	976(63.87)	552(36.13)	0.306931	313(40.97)	350(45.81)	101(13.22)	0.712871	0.839
	CTR	1355(71.92)	529(28.08)		485(51.49)	385(40.87)	72(7.64)		0.714	2972(65.18)	1588(34.82)		970(42.54)	1032(45.26)	278(12.19)		0.890
rs3774601		G	A		GG	GA	AA			G	A			GG	GA	AA	
F	SCZ	889(76.51)	273(23.49)	0.197225	337(58)	215(37.01)	29(4.99)	0.305520	0.479	1071(80.41)	261(19.59)	0.118812	429(64.41)	213(31.98)	24(3.6)	0.475248	0.699
	CTR	1299(74.4)	447(25.6)		486(55.67)	327(37.46)	60(6.87)		0.621	3175(78.78)	855(21.22)		1251(62.08)	673(33.4)	91(4.52)		0.968
M	SCZ	834(77.8)	238(22.2)	0.144664	319(59.51)	196(36.57)	21(3.92)	0.148778	0.175	1252(81.94)	276(18.06)	0.297030	508(66.49)	236(30.89)	20(2.62)	0.336634	0.228
	CTR	1421(75.42)	463(24.58)		538(57.11)	345(36.62)	59(6.26)		0.711	3680(80.7)	880(19.3)		1485(65.13)	710(31.14)	85(3.73)		0.991
rs3774605		C	T		CC	CT	TT			C	T			CC	CT	TT	
F	SCZ	1013(87.18)	149(12.82)	0.250920	443(76.25)	127(21.86)	11(1.89)	0.522391	0.591	1214(91.14)	118(8.86)	0.207921	553(83.03)	108(16.22)	5(0.75)	0.356436	0.913
	CTR	1496(85.68)	250(14.32)		643(73.65)	210(24.05)	20(2.29)		0.562	3627(90)	403(10)		1632(80.99)	363(18.01)	20(0.99)		0.970
M	SCZ	919(85.73)	153(14.27)	0.245832	390(72.76)	139(25.93)	7(1.31)	0.183638	0.167	1377(90.12)	151(9.88)	0.415842	624(81.68)	129(16.88)	11(1.44)	0.267327	0.151
	CTR	1585(84.13)	299(15.87)		669(71.02)	247(26.22)	26(2.76)		0.579	4078(89.43)	482(10.57)		1821(79.87)	436(19.12)	23(1.01)		0.584

SCZ: schizophrenia; CTR: control

1. p-values of the normal chi-square statistics from Monte Carlo stimulation using CLUMP (T2).

Table S3. Summary of the association test for imputed and typed SNPs using frequentist method.

typed	rs ID	chromosome	position	alleleA	alleleB	MAF	p-value
imputed	rs17053026	3	53393838	C	T	0.021803	0.965601
imputed	rs7630080	3	53495180	A	G	0.173381	0.145435
imputed	rs312480	3	53504180	C	T	0.150914	0.0544111
imputed	rs312487	3	53520662	C	T	0.428969	0.0260372
imputed	rs979220	3	53531263	C	T	0.277493	0.630679
imputed	rs3774430	3	53541490	A	G	0.144803	0.772245
imputed	rs9821489	3	53550768	A	G	0.18641	0.668399
imputed	rs12715461	3	53553923	A	G	0.364162	0.0766316
imputed	rs6445584	3	53562884	A	G	0.052615	0.25819
imputed	rs2359453	3	53567904	A	G	0.366846	0.0593187
imputed	rs17053186	3	53575177	C	T	0.231268	0.538227
imputed	rs17053195	3	53579995	A	G	0.038559	0.744416
imputed	rs11707155	3	53583346	A	G	0.017884	0.0130713
imputed	rs709317	3	53584650	A	G	0.170271	0.310384
imputed	rs709318	3	53584861	C	T	0.037522	0.522151
imputed	rs17053206	3	53584940	C	T	0.037584	0.729103
genotyped	rs709323	3	53589524	A	C	0.21476	0.212286
imputed	rs807198	3	53590498	A	T	0.389382	0.201553
imputed	rs807197	3	53590738	C	T	0.183199	0.166577
imputed	rs807194	3	53591110	A	C	0.285451	0.147553
imputed	rs807193	3	53591378	A	G	0.255971	0.115306
imputed	rs807192	3	53591641	C	G	0.034697	0.469035
genotyped	rs3774458	3	53592908	A	G	0.164454	0.230389
imputed	rs3774463	3	53598142	C	T	0.095695	0.472525
imputed	rs6445588	3	53605719	C	T	0.339507	0.527596
imputed	rs936450	3	53609466	A	G	0.496221	0.162494
imputed	rs9866078	3	53610482	A	G	0.462212	0.212784
imputed	rs9810888	3	53610635	G	T	0.457997	0.357922
imputed	rs7610071	3	53612735	C	T	0.333987	0.253543
imputed	rs17309406	3	53613751	G	T	0.064286	0.103387
imputed	rs3774473	3	53613891	A	G	0.161939	0.624262
imputed	rs11721212	3	53614979	C	T	0.042744	0.0380448
imputed	rs11918955	3	53616124	A	G	0.15867	0.799889
imputed	rs17053265	3	53621579	A	G	0.056947	0.535329
imputed	rs1460115	3	53622852	C	T	0.216633	0.349847
imputed	rs6794085	3	53624648	A	G	0.182652	0.122593
imputed	rs13063116	3	53624717	A	G	0.1259	0.531759
imputed	rs3796345	3	53628192	C	T	0.308859	0.069198
imputed	rs3774480	3	53629933	C	T	0.24098	0.333563
imputed	rs9850569	3	53631844	A	G	0.444539	0.106372
imputed	rs17053279	3	53634757	A	G	0.049128	0.495075
imputed	rs12493744	3	53635070	C	T	0.379856	0.999223

imputed	rs3774487	3	53637234	A	G	0.16333	0.478643
imputed	rs3821854	3	53638332	C	T	0.460129	0.19373
imputed	rs3774490	3	53638688	C	T	0.130673	0.312897
imputed	rs3774491	3	53638875	C	G	0.30284	0.602494
imputed	rs6802110	3	53642272	A	G	0.06092	0.0191367
imputed	rs898415	3	53642679	A	G	0.233256	0.231991
imputed	rs1380606	3	53643172	C	T	0.232981	0.228397
imputed	rs11707976	3	53643748	A	G	0.091172	0.12223
imputed	rs11709171	3	53644944	A	C	0.032384	0.0133293
imputed	rs6786135	3	53645005	C	T	0.30216	0.572377
imputed	rs2077167	3	53646459	C	T	0.442368	0.137004
imputed	rs6763245	3	53650755	A	G	0.369521	0.0305456
imputed	rs6803827	3	53651373	A	G	0.491905	0.107498
imputed	rs3774503	3	53652225	A	G	0.127859	0.541786
imputed	rs1037827	3	53658352	A	G	0.442722	0.47058
imputed	rs2276836	3	53660079	C	T	0.379868	0.024254
imputed	rs11712998	3	53663998	A	G	0.088699	0.113746
imputed	rs3774519	3	53670282	G	T	0.072519	0.0197056
imputed	rs9881913	3	53671328	A	G	0.027275	0.648157
imputed	rs2250736	3	53675590	C	T	0.283242	0.377957
imputed	rs3821856	3	53675744	A	G	0.071161	0.184858
imputed	rs3774523	3	53675800	C	T	0.052977	0.436216
imputed	rs3774527	3	53680043	C	T	0.018084	0.852718
imputed	rs11130376	3	53680390	C	T	0.025625	0.0597129
imputed	rs2633707	3	53684122	C	G	0.288959	0.116806
genotyped	rs1460118	3	53684891	C	T	0.272314	0.353417
imputed	rs1380609	3	53689216	C	T	0.097898	0.204061
imputed	rs12492502	3	53690114	A	G	0.320555	0.0739116
imputed	rs2680672	3	53691013	C	T	0.44657	0.185455
imputed	rs898411	3	53691952	C	T	0.286279	0.330672
imputed	rs898422	3	53692322	A	G	0.190529	0.340744
imputed	rs2612018	3	53694125	C	T	0.142946	0.47732
imputed	rs11130377	3	53697914	C	T	0.276576	0.821881
imputed	rs1380608	3	53699410	A	G	0.10837	0.999516
imputed	rs898417	3	53704431	C	T	0.05879	0.365498
imputed	rs9841978	3	53705775	A	G	0.28915	0.618174
imputed	rs7340705	3	53709483	C	T	0.404019	0.958748
imputed	rs6445597	3	53709571	A	G	0.293921	0.496198
imputed	rs9830632	3	53710806	A	G	0.269586	0.549856
imputed	rs17053363	3	53711597	A	T	0.075801	0.142328
imputed	rs3774529	3	53712201	G	T	0.076296	0.000974805
genotyped	rs3774530	3	53712410	C	T	0.36	0.132757
imputed	rs3774531	3	53712615	C	T	0.463788	0.0534673
imputed	rs3774533	3	53713360	A	G	0.394867	0.0329672
imputed	rs2633731	3	53713464	C	T	0.25548	0.100647

imputed	rs3821857	3	53713500	C	T	0.092763	0.59609
imputed	rs1380605	3	53713814	C	T	0.412203	0.0299649
imputed	rs2633730	3	53715739	C	T	0.489768	0.0017902
imputed	rs2633728	3	53715979	C	G	0.186695	0.126448
imputed	rs3774541	3	53716172	A	G	0.32014	0.00714984
imputed	rs2680661	3	53717188	G	T	0.396977	0.0117334
imputed	rs2612015	3	53717371	G	T	0.191034	0.570121
imputed	rs6445598	3	53717776	A	T	0.136991	0.0956017
imputed	rs2633727	3	53721129	A	G	0.041382	0.109192
imputed	rs10510765	3	53721246	C	G	0.062964	0.0291484
imputed	rs13076366	3	53722414	A	C	0.112568	0.0701254
imputed	rs11708227	3	53723073	C	T	0.113247	0.0712147
imputed	rs11709505	3	53724538	C	T	0.113298	0.0729244
imputed	rs2633725	3	53726782	C	T	0.051185	0.120158
imputed	rs2070617	3	53733089	A	G	0.339687	0.533793
imputed	rs7638857	3	53733363	A	C	0.449064	0.427743
imputed	rs7649275	3	53733743	A	G	0.051643	0.170536
imputed	rs17053417	3	53734429	A	G	0.140852	0.693104
imputed	rs11719179	3	53734761	A	G	0.069926	0.0757988
imputed	rs1020819	3	53737753	A	C	0.477101	0.695866
imputed	rs2612033	3	53741066	C	T	0.051629	0.170861
imputed	rs2029331	3	53742729	C	G	0.172573	0.641362
imputed	rs17030975	3	53743245	C	T	0.412553	0.546145
imputed	rs1045958	3	53744519	C	T	0.215696	0.0771334
imputed	rs898423	3	53744805	A	G	0.231662	0.356364
imputed	rs6800641	3	53745628	A	G	0.153385	0.823146
imputed	rs2612028	3	53750487	C	T	0.123844	0.0452602
imputed	rs2612027	3	53751718	A	G	0.483107	0.00575574
imputed	rs2612026	3	53752035	C	T	0.161979	0.117266
imputed	rs2680648	3	53752216	C	T	0.202035	0.0257444
imputed	rs2612025	3	53753354	C	T	0.162663	0.133273
imputed	rs2253795	3	53753915	G	T	0.124942	0.0474546
imputed	rs3821860	3	53754865	A	G	0.194736	0.793547
imputed	rs17053459	3	53756034	A	G	0.062464	0.0248007
imputed	rs1809282	3	53756997	A	G	0.174244	0.114866
imputed	rs719260	3	53764212	C	T	0.202151	0.0293601
imputed	rs17053476	3	53764611	C	T	0.054917	0.830119
imputed	rs7373253	3	53764634	C	T	0.047577	0.166375
imputed	rs3774563	3	53765316	A	G	0.324104	0.38218
imputed	rs4687738	3	53765449	A	G	0.031756	0.681566
imputed	rs1401497	3	53766496	A	G	0.374156	0.37483
imputed	rs755886	3	53766988	A	T	0.076469	0.325609
imputed	rs12487452	3	53767451	C	G	0.428995	0.123964
imputed	rs3796347	3	53767930	A	G	0.38744	0.709586
imputed	rs3774573	3	53775000	C	T	0.226126	0.0640128

imputed	rs1109959	3	53775262	C	T	0.081633	0.084326
imputed	rs3774577	3	53777303	C	T	0.214881	0.847773
imputed	rs3821865	3	53777335	C	T	0.483138	0.038997
imputed	rs3774581	3	53777788	A	G	0.333624	0.0241198
imputed	rs3774586	3	53786346	A	G	0.468061	0.0526836
imputed	rs7617553	3	53788272	A	G	0.488338	0.00935734
imputed	rs1829423	3	53789237	A	G	0.481822	0.0158282
imputed	rs11707420	3	53790905	A	G	0.457621	0.0391082
imputed	rs3774589	3	53792398	G	T	0.471245	0.0331647
imputed	rs3774598	3	53797359	A	G	0.060806	0.579825
genotyped	rs3774601	3	53797933	A	G	0.198428	0.0976903
imputed	rs3774602	3	53798138	C	T	0.451118	0.00490801
imputed	rs3774604	3	53799176	C	T	0.173542	0.147659
genotyped	rs3774605	3	53805807	A	G	0.100786	0.167836
imputed	rs3774608	3	53807752	A	G	0.489967	0.00588698
imputed	rs3774609	3	53807943	G	T	0.48872	0.00771914
imputed	rs3796349	3	53808549	A	G	0.241296	0.00131746
imputed	rs2359133	3	53811782	C	G	0.224395	0.326069
imputed	rs6766988	3	53814510	A	T	0.186663	0.532592
imputed	rs3774611	3	53815705	A	G	0.424272	0.0149894
imputed	rs7620619	3	53816844	G	T	0.040256	0.52209
imputed	rs10154841	3	53820335	C	T	0.236889	0.23392
imputed	rs10490844	3	53821117	A	G	0.084389	0.20107
imputed	rs877483	3	53821781	C	T	0.255213	0.811308
imputed	rs877484	3	53821964	A	G	0.468123	0.155127
imputed	rs893363	3	53822102	A	G	0.244722	0.107912
imputed	rs4687587	3	53824550	A	G	0.045688	0.000857143
imputed	rs7625247	3	53829108	G	T	0.423125	0.323176
imputed	rs9836592	3	53830123	C	T	0.149111	0.0139736
imputed	rs7626693	3	53833581	C	T	0.469813	0.29628
imputed	rs13317328	3	53845880	A	C	0.294388	0.0122726
imputed	rs6445607	3	53852189	G	T	0.302292	0.539262
imputed	rs3774616	3	53852973	C	T	0.201335	0.0139659
imputed	rs1025690	3	53857703	A	G	0.11246	0.0442932
imputed	rs999514	3	53864889	C	T	0.245371	0.0065726
imputed	rs2232344	3	53866727	A	G	0.010864	0.870174
imputed	rs17053571	3	53876834	C	T	0.171024	0.0212077
imputed	rs2289204	3	53881374	A	C	0.179411	0.0214243
imputed	rs2276847	3	53900815	C	T	0.170387	0.0280084
imputed	rs7636856	3	53909211	A	G	0.21158	0.00484205
imputed	rs7624521	3	53923356	G	T	0.361294	0.00433826
imputed	rs11130386	3	53968526	A	G	0.17175	0.0482104

Table S4 General power analysis of testing dataset

OR \ MAF	0.05	0.10	0.20	0.30	0.40	0.50
1.1	0.107	0.147	0.184	0.182	0.159	0.127
1.2	0.272	0.414	0.525	0.512	0.434	0.324
1.3	0.505	0.716	0.829	0.810	0.719	0.562
1.4	0.727	0.905	0.961	0.950	0.894	0.758
1.5	0.878	0.978	0.994	0.991	0.968	0.882

OR: odds ratio; MAF: minor allele frequency

Table S5 General power analysis of validation dataset

OR \ MAF	0.05	0.10	0.20	0.30	0.40	0.50
1.1	0.158	0.233	0.301	0.296	0.252	0.193
1.2	0.461	0.668	0.789	0.773	0.682	0.530
1.3	0.783	0.938	0.979	0.972	0.932	0.817
1.4	0.948	0.995	0.999	0.999	0.992	0.949
1.5	0.993	0.999	0.999	0.999	0.999	0.989

OR: odds ratio; MAF: minor allele frequency
